# How does the canine paw pad attenuate ground impacts? A multi-layer cushion system

**DOI:** 10.1242/bio.024828

**Published:** 2017-11-23

**Authors:** Huaibin Miao, Jun Fu, Zhihui Qian, Luquan Ren, Lei Ren

**Affiliations:** 1 Key Laboratory of Bionic Engineering, Jilin University, Changchun 130022, People's Republic of China; 2 School of Mechanical, Aerospace and Civil Engineering, University of Manchester, Manchester M13 9PL, UK

**Keywords:** Paw pad, Micro-scale, Cushioning, Multi-layer

## Abstract

Macroscopic mechanical properties of digitigrade paw pads, such as non-linear elastic and variable stiffness, have been investigated in previous studies; however, little is known about the micro-scale structural characteristics of digitigrade paw pads, or the relationship between these characteristics and the exceptional cushioning of the pads. The digitigrade paw pad consists of a multi-layered structure, which is mainly comprised of a stratified epithelium layer, a dermis layer and a subcutaneous layer. The stratified epithelium layer and dermal papillae constitute the epidermis layer. Finite element analyses were carried out and showed that the epidermis layer effectively attenuated the ground impact across impact velocities of 0.05–0.4 m/s, and that the von Mises stresses were uniformly distributed in this layer. The dermis layer encompassing the subcutaneous layer can be viewed as a hydrostatic system, which can store, release and dissipate impact energy. All three layers in the paw pad work as a whole to meet the biomechanical requirements of animal locomotion. These findings provide insights into the biomechanical functioning of digitigrade paw pads and could be used to facilitate bio-inspired, ground-contacting component development for robots and machines, as well as contribute to footwear design.

## Abstract (precis)

**Summary:** This study examines the micro-scale structural characteristics of the digitigrade paw pad and analyses how these structures work together to further clarify the paw pad cushioning mechanism.

## INTRODUCTION

The feet of digitigrade mammals (e.g. dogs and cats) are different anatomically from the feet of plantigrades (Boyd et al., 2001). Digitigrades have relatively long carpals and tarsals as well as large metacarpal pads, and the paw pads located beneath the digits and metacarpals are in contact with the ground's surface during locomotion. Biewener (1990) found that animals generally exert ground reaction forces (GRFs) at two to three times their body weight per limb. Additionally, digitigrades generally move more quickly than other animals with footpads (Minetti, 2000; Knight, 2012). Thus, their paw pads may be subjected to larger transient GRFs, as high as six times their body weight (Alexander et al., 1986). A shockwave caused by GRF is usually transmitted along the body and reaches the skull. It has been suggested that GRFs, and the related shockwaves, are the primary etiological agents in degenerative joint diseases and musculoskeletal system injuries (Collins and Whittle, 1989; Whittle, 1999; Gill and O'Connor, 2003a,b; Chi and Schmitt, 2005; Jahss et al., 1992). The digitigrade paw pad is comprised of digital and metacarpal pads, which are usually located under distal interphalangeal joints and metacarpophalangeal joints, respectively (Boyd et al., 2001; Hubbard et al., 2009). It is believed that paw pads attenuate foot-ground impact forces and protect the musculoskeletal system since they are the only body components in contact with the ground (Whittle, 1999).

Over the past decades, a large number of studies have been conducted to investigate the footpad biomechanics of land mammals; however, most of those studies have focused on the mechanical behaviour of heel pads in plantigrades. Erdemir et al. (2006) investigated and showed the importance of subject-specific modelling of the nonlinear elastic behaviour of the heel pad. Ledoux and Blevins (2007) determined the material properties of the plantar soft tissue and identified differences between the subcalcaneal tissue and other plantar soft tissue areas. Natali et al. (2010) analysed experimental data from mechanical tests and presented a visco-hyperelastic constitutive model to describe the biomechanical response of heel pad tissues. Qian et al. (2010) revealed impact attenuation and energy absorption functions of the human foot complex based on *in vivo* gait measurements, finite element modelling and biological coupling theory. Fontanella et al. (2013) investigated the interactions occurring between the foot and footwear during the heel strike phase of the gait and found compressive stress differentiation of the heel pad region between bare and shod conditions. Nevertheless, there have been few studies on the biomechanical behaviour of digitigrade paw pads (Chi, 2005). Alexander et al. (1986) examined the dynamic elastic properties of the paw pads of some mammals, including the red-necked wallaby (*Macropus rufogriseus*), badger (*Meles meles*), domestic dog (*Canis fumiliuris*), red fox (*Vulpes vulpes*), domestic cat (*Felis catus*) and Arabian camel (*Cumelus dromedarius*), and found that all have non-linear elastic properties, with energy dissipations of 15–43%. Chi and Roth (2010) found that the structural properties (structural properties of an object are a consequence of its size, geometry and material) of animal footpads, unlike other biological supporting structures that have constant material properties, scale in proportion to body mass by changing both their geometry and material properties in order to maintain and operate the musculoskeletal locomotor system. However, only the visco-elastic and scaled stiffness properties of whole digitigrade paw pads were examined, unlike plantigrade footpads, which were systematically studied. Studies of the internal structural characteristics of digitigrade paw pads are lacking, limiting our understanding of paw pad cushion mechanisms.

The objective of this study was to investigate the biomechanical cushion mechanism of canine paw pads on a micro-scale level. A German Shepherd dog (*Canis lupus familiaris*) and Chinese Rural dog (*Canis lupus familiaris*) were studied. The German Shepherd is one of the most popular breeds of dogs worldwide and is often the preferred breed for many types of work due to their strong locomotion capabilities. The Chinese Rural dog is the most widely distributed breed in China and can adapt to a wide variety of ground conditions. Histological examination of paw pad tissues was conducted via tissue staining and scanning electron microscopy (SEM). Micromechanical analysis, based on the finite element (FE) method, was used to investigate the relationship between microstructure and biomechanical functioning.

## RESULTS

### Scanning electron microscopy

**Fig. 1.**
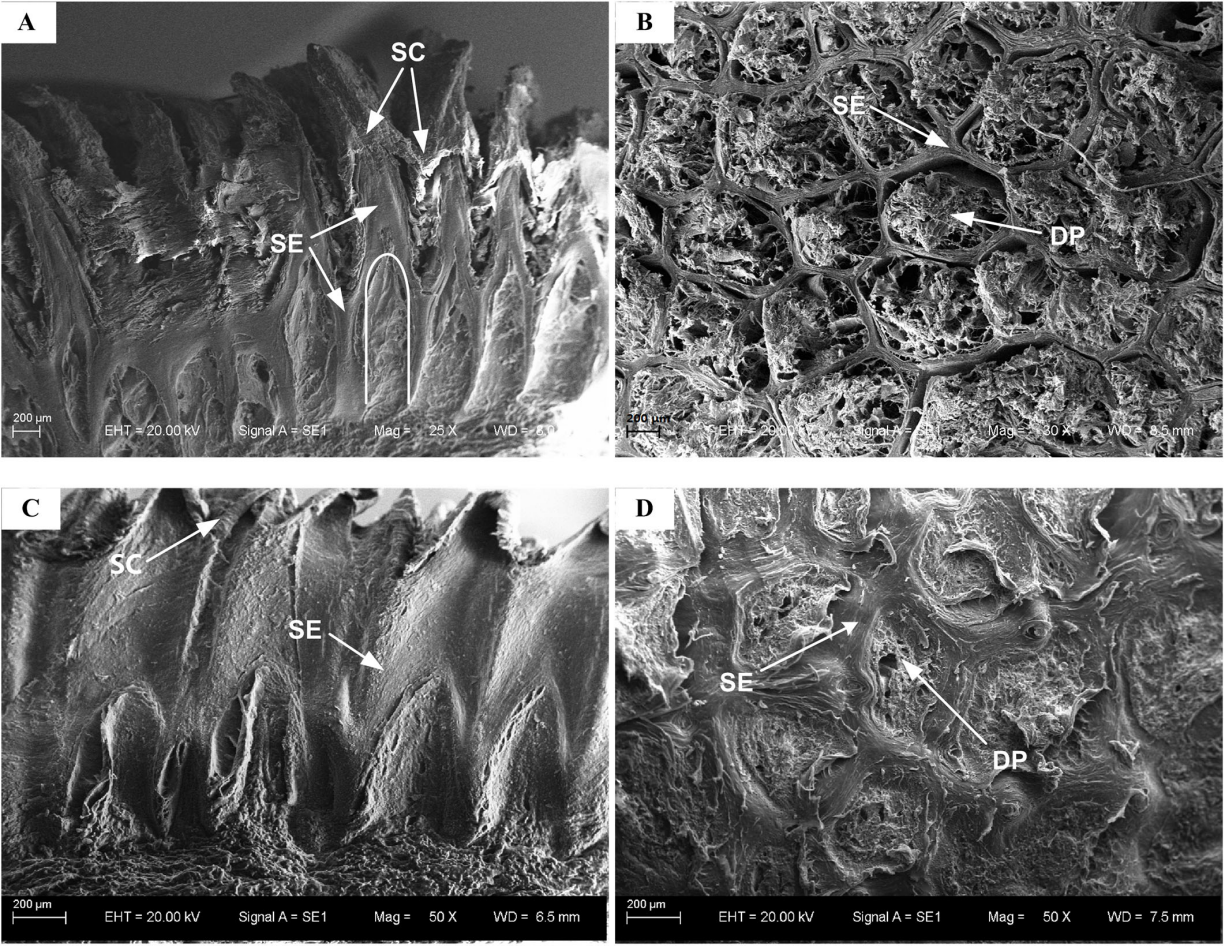
**Scanning electron microscope images of the stratified epithelium.** (A) Sagittal section of the stratified epithelium of a German Shepherd dog. White line represents the profile of honeycomb crust. (B) Transverse section of the stratified epithelium of a German Shepherd dog. (C) Sagittal section of the stratified epithelium of a Chinese Rural dog. (D) Transverse section of the stratified epithelium of a Chinese Rural dog. SC, stratum corneum; SE, stratified epithelium; DP, dermal papillae.

The epidermis layer of the paw pad was investigated using a SEM. The results are shown in Fig. 1. Many spike-like structures were distributed on the outside of the epidermis layer and many pits were present on the inside of the epidermis layer. The ratio of the pits to the spike-like structures was 1:1 (Fig. 1A,C). The cross-section contained many holes, arranged in a honeycomb-like fashion (Fig. 1B,D). The epidermis layer mainly consisted of a stratified epithelium layer and a stratum corneum layer (Fig. 1A,C). The holes were embedded with dermal papillae (Fig. 1B,D). The paw pads of both the German Shepherd dog and Chinese Rural dog had similar structures.

### Histology

**Fig. 2.**
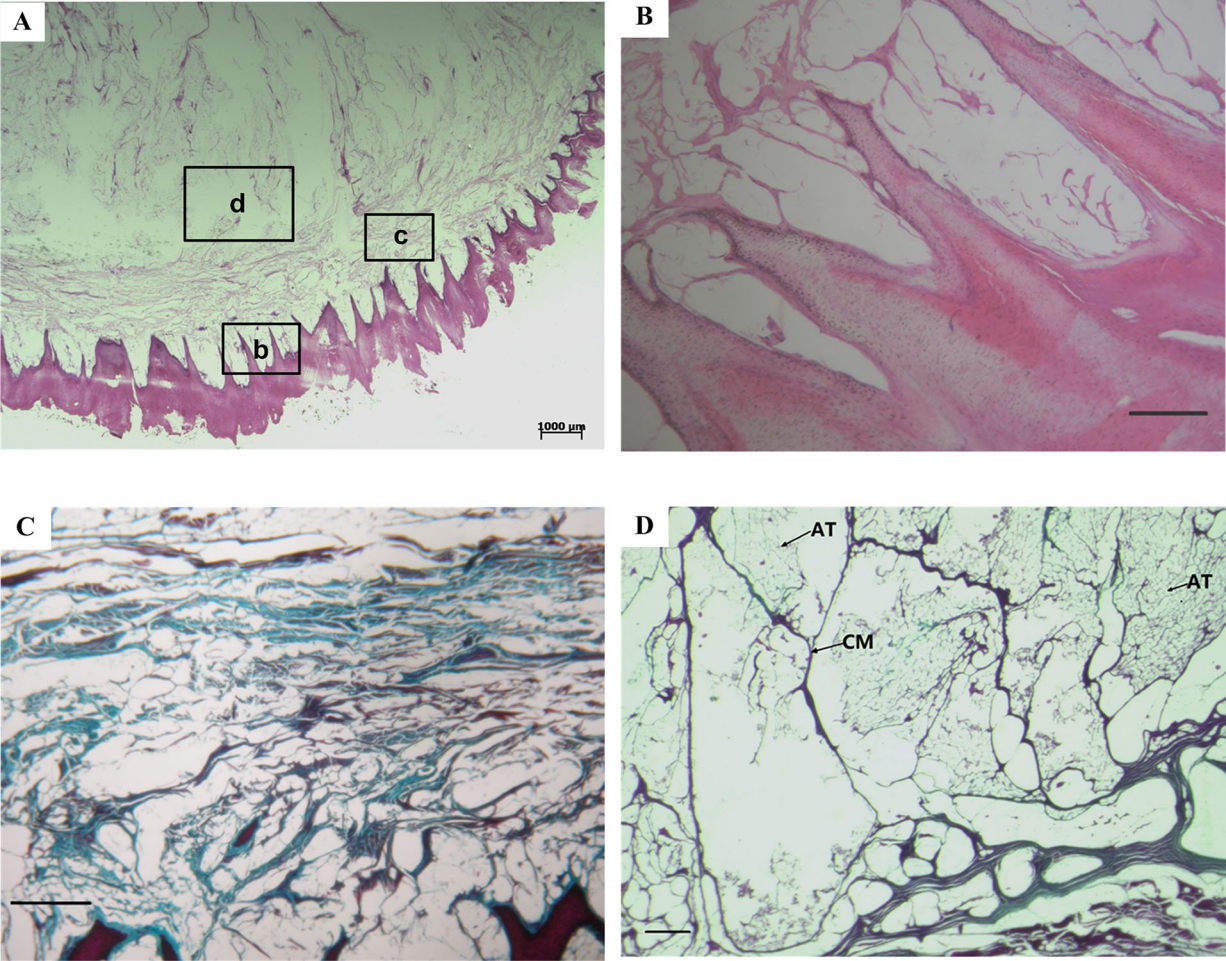
**Histological images of the paw pad.** (A) Whole mount of the paw pad. (B) Stratified epithelium and dermal papillae, b region in panel A. (C) Dermis layer, c region in panel A. Collagen fibres are in blue. (D) Collagenous membranes and adipose tissue distributed in the subcutaneous adipose tissue, d region in panel A. CM, collagenous membrane; AT, adipose tissue. A and B were stained with hematoxylin and eosin; C and D were stained with Masson. Scale bars in B, C and D represent 200 μm.

Histological examination results of paw pad samples from a representative dog are shown in Fig. 2. The bottom surface of the paw pad, which was in direct contact with the ground surface during locomotion, is covered by a layer of spike-like stratum corneum (Fig. 2A). The dermal papillae are composed of matrix tissues and are basically small protrusions of the dermis projecting into the honeycomb cells of the stratified epithelium (Fig. 2A,B). Interestingly, this honeycomb-like structure was only found in the bottom epidermis layer of the paw pad that was in contact with the ground. The epidermis of the other parts of the footpad (e.g. the layer by the side wall) does not show the structured honeycomb pattern (Fig. S1). Further up, superiorly is the dermis layer, which lies adjacent to the stratified epithelium and dermal papillae. There is a reticulated layer of collagen fibre bundles, with a few interspersed elastic fibres distributed in the middle of the dermis layer (Fig. 2C). The subcutaneous layer, containing a large amount of subcutaneous adipose tissue, is above the dermis layer (Fig. 2A) and is separated into many small compartments by collagenous membranes (Fig. 2D).

### Finite element analysis

**Fig. 3.**
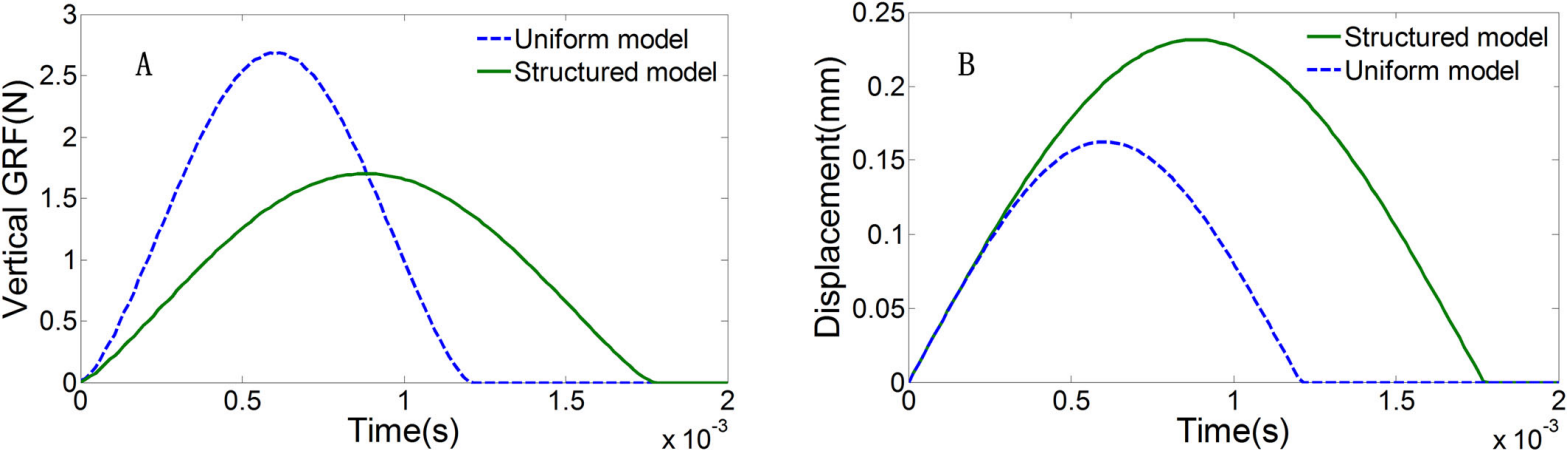
**The predicted time histories by both the uniform model and the structured model.** (A) Vertical GRF; (B) the displacements of top plates.

To investigate the mechanical functioning of the characteristic honeycomb-structured epidermis layer, dynamic FE simulations were conducted at five different initial loading velocities, using both the structured model and the uniform models. Fig. 3 shows the simulated vertical GRFs and the vertical displacements of the top plate in both models under a loading velocity of 0.4 m/s. The GRFs and vertical displacements show typical single-hump patterns associated with a loading and unloading cycle. From Fig. 3A, it can be seen that the structured epidermis layer lowers the peak vertical GRF acting on the paw pad leading to a 37% drop in peak force from 2.7 N to 1.7 N, in contrast to the uniform model. The peak vertical displacement of the top mass increases by about 42% in the structured model (Fig. 3B). The contact time between the model and the ground was longer (0.0009 s) with the structured model compared to the uniform model (0.0006 s).

**Fig. 4.**
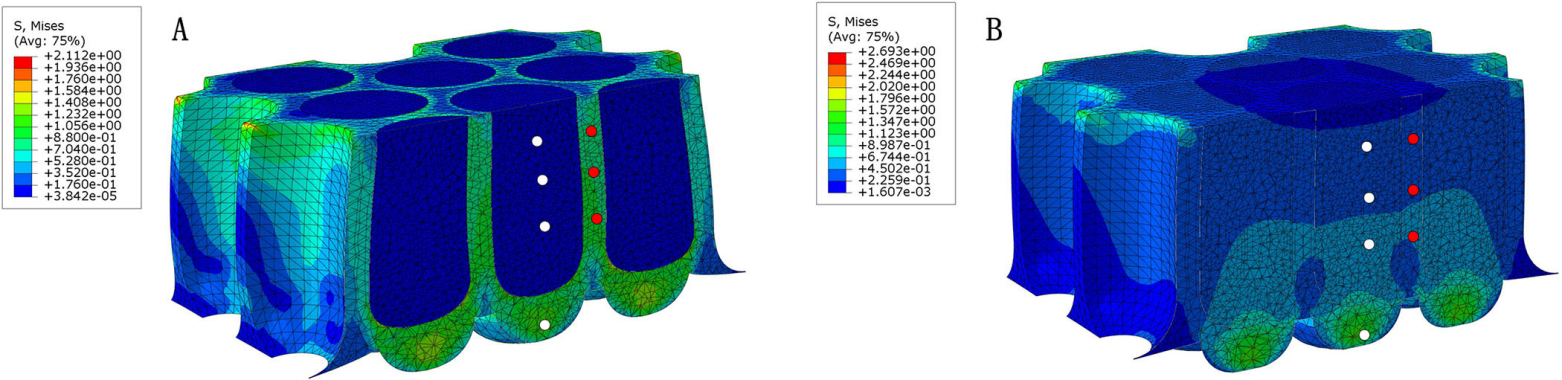
**The simulated von Mises stress distribution under peak GRFs at an impact velocity of 0.4 m/s.** (A) Structured model; (B) uniform model.

The von Mises stresses, which were simulated in both models under the peak vertical GRFs, are shown in Fig. 4. In the structured model, the von Mises stress drops dramatically along the longitudinal axis of the dermal papillae from bottom to top (see the four white nodes in Fig. 4A with von Mises stresses of 1.218, 0.00077, 0.00075, and 0.00032 MPa, respectively in a bottom-up order). Whereas in the uniform model, higher von Mises stresses are shown at the same nodes along the longitudinal axis, also in a bottom-up decreasing pattern (see the four white nodes in Fig. 4B with von Mises stresses of 1.393, 0.51, 0.42 and 0.3 MPa, respectively). However, the stress decreases at a slower rate than in the structured model. Interestingly, the opposite trend is seen in with simulated stresses in the stratified epithelium. The structured model predicts higher von Mises stresses than the uniform model at the same nodes (the three red nodes in Fig. 4A with von Mises stresses of 0.916, 0.865, and 0.736 MPa from bottom to top and the corresponding red nodes in Fig. 4B with von Mises stresses of 0.448, 0.433, and 0.334 MPa from bottom to top). Therefore, the bottom-up decreasing stress pattern along the longitudinal direction is present in the stratified epithelium in both models. However, the grade of stress decrease is lower in the structured than in the uniform model. In addition, the distribution of von Mises stresses is much larger in the region that is in contact with the ground in the uniform than in the structured model. The von Mises stresses uniformly distribute in the stratified epithelium layer in the structured model, which may effectively circumvent stress concentration.

**Fig. 5.**
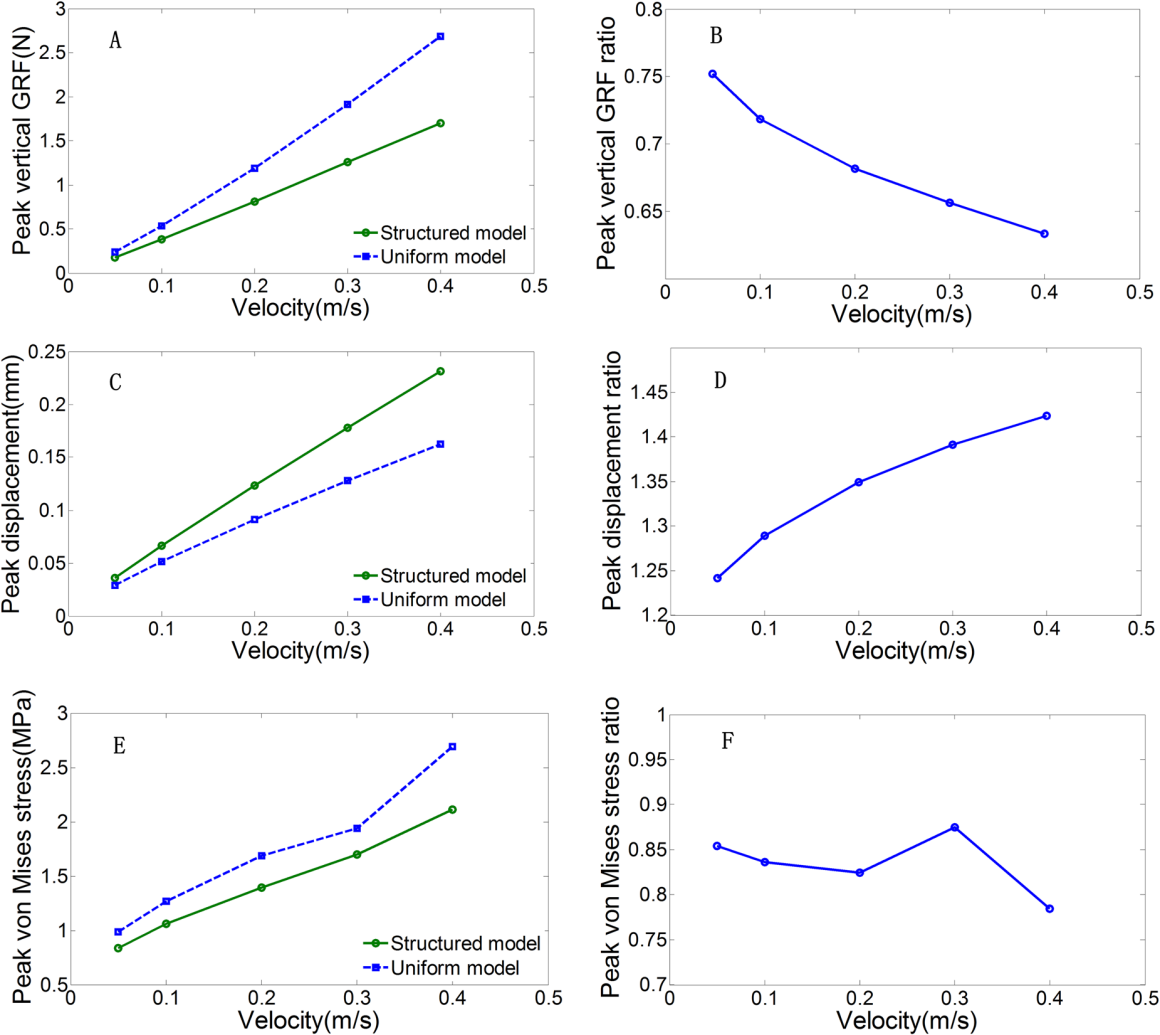
**The FE simulations results of the structured and uniform models with an initial velocity ranging from 0.05 m/s to 0.4 m/s.** The peak vertical GRFs (A), the peak vertical displacements (C) and the peak von Mises stresses (E) predicted by the structured and uniform models at different impact velocities. The peak vertical GRF ratio (B), the peak vertical displacement ratio (D) and the peak von Mises stress ratio (F) between the structured model and the uniform model across different impact velocities.

Peak vertical GRF is the primary output variable in this study. The peak vertical GRF increased from 0.18 N to 1.7 N in the structured model and from 0.24 N to 2.69 N in the uniform model when the impact velocity changed from 0.05 m/s to 0.4 m/s (Fig. 5A). The increase in slope was greater in the uniform model than in the structured model as demonstrated by the ratios of the peak GRF predicted by the structured model versus those predicted by the uniform model at different impact velocities (Fig. 5B). The peak vertical GRF ratios decreased from 0.75 to 0.63 with as impact velocities increased. This indicates that the structured model had a better capacity for peak vertical GRF attenuation and cushioning, especially at higher impact velocities, compared with the uniform model.

Similar to the vertical peak GRF, the peak vertical displacements of the top plates increased with increasing impact velocity in both models (Fig. 5C). However, the peak vertical displacements in the structured model (0.04, 0.07, 0.12, 0.18, 0.23 mm) were larger than in the uniform model (0.03, 0.05, 0.09, 0.13, and 0.16 mm) at different impact velocities. This can be seen from the peak displacement ratios between the two models at different impact velocities (Fig. 5D). The peak displacement ratios increased from 1.24 to 1.42 with increasing impact velocities. The maximum von Mises stresses increased from 0.839 MPa to 2.112 MPa in the structured model and from 0.983 MPa to 2.693 MPa in the uniform model with increasing impact velocities (Fig. 5E). Obviously, the structured model generated lower maximum von Mises stresses than the uniform model. The maximum von Mises stress ratios of the two models at different impact velocities are shown in Fig. 5F. Overall, the ratios decreased with increasing impact velocities, except at a speed of 0.3 m/s.

**Fig. 6.**
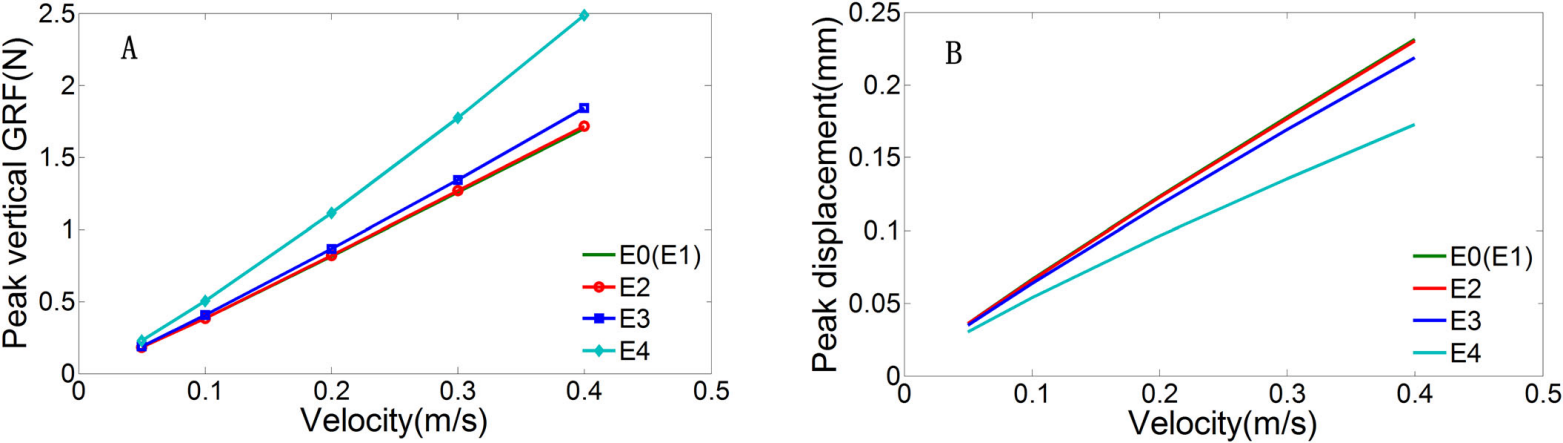
**The simulated peak vertical GRFs and peak vertical displacements by the structured model across different impact velocities with Young's modulus of the dermal papilla set at different values E1=0.1 E0, E2=10 E0, E3=100 E0, E4=1000 E0, where E0=0.004 MPa.** (A) Peak vertical GRFs; (B) peak vertical displacements of top plates.

Material property sensitivity analysis was used to investigate the effect of the Young's modulus of the dermal papilla on peak vertical GRFs and on peak vertical displacements at the top plate at different impact velocities. The results of the material property sensitivity analysis are shown in Fig. 6. There was an almost linear increase in peak vertical GRFs and vertical displacements with increasing impact velocity. A lower slope in the peak vertical GRF curve and a higher gradient in the peak vertical displacement curve were observed with a softer dermal papilla material. This indicates that the decrease in the Young's modulus of the dermal papilla greatly attenuated peak vertical GRF, especially at higher impact velocities; however, this increase in cushioning capacity plateaued. When the Young's modulus of the dermal papilla was lower than a critical value of 0.04-0.004 MPa, the slopes of both the peak vertical GRF and peak vertical displacement curves stopped increasing as the Young's modulus decreased. In this scenario, the cushioning function of the epidermis layer reached maximum capacity.

## DISCUSSION

The SEM and histological examination results suggest that the paw pads of the German Shepherd dog and the Chinese Rural dog are mainly comprised of three layers: the external stratified epithelium, the intermediate dermis layer and the subcutaneous layer. Among the three layers, the external stratified epithelium layer is composed of the hardest material and is in direct contact with the ground. The stratified epithelium layer, along with the outermost stratum corneum layer, is subjected to tremendous pad-ground wear, friction and impact during locomotion (Meyer et al., 1990). The subcutaneous layer consists of adipose tissue, which is basically adipocytes filled with lipids. The adipose tissue is separated into many small compartments by collagenous membranes. Similar structures are also found in the subcutaneous layer of the footpads of human beings, elephants, cats and leopards (Alexander et al., 1986; Weissengruber et al., 2006; Hubbard et al., 2009; Qian et al., 2010; Mihai et al., 2015). The mechanical behaviour of adipose tissue is considered to be equivalent to a hydrostatic system filled with incompressible fluid (Pond, 1998; Ker, 1999; Chi and Roth, 2010). Based on this, the dermis layer encompassing the subcutaneous layer can be regarded as a large hydrostatic system. Indeed, of the three layers, the subcutaneous layer is composed of the softest material and is the foremost energy absorber of footpads (Ker, 1999; Weissengruber et al., 2006). The dermis layer lies between the hardest and softest layers of the footpad, of which the Young's modulus has about 6000 times difference between the hardest and softest materials. Thus far, little is known about the biomechanical functioning of the dermis during locomotion. In this study, we found that the dermis layer is composed of two parts: dermal papillae, and another part that is rich in collagen fibres. The dermal papillae and the stratified epithelium layer, which have distinctive honeycomb-like structures at the micro-scale level, make up the epidermis layer; this structure is also found in the paw pad of the Beagle dog (Ninomiya et al., 2011) and in the paw pad of the Clouded leopard (Hubbard et al., 2009). Therefore, it appears that this special micro-structured layer is common in the paw pads of digitigrades, and to the best of our knowledge, has not been studied previously. The dynamic FE analyses in the current study indicate that this special micro-structured layer may have multiple biomechanical functions.

Cushioning is one of the most important biomechanical functions of feet. The FE simulation results of this study showed that the structured epidermis layer attenuates peak GRF across a range of impact velocities much more effectively than the uniform epithelium layer. This is not surprising since the dermal papillae, which fill the cell units of a honeycomb-like structure, are much softer than epithelial tissue. Interestingly, the structured epidermis layer also confers favourable mechanical characteristics to the footpad by providing stronger cushioning capacity at higher impact velocities. The simulation results of the current study suggest that peak GRF increases almost linearly with increasing impact velocity; thus, a strong cushioning capacity is highly desirable for the locomotor system. Because both the stratified epithelium and the dermal papillae were modelled as linear-elastic materials, rather than visco-elastic materials in this study, this velocity-dependent cushioning capacity is very likely due to the honeycomb-like structure at the micro-scale level. Moreover, the cushioning capacity of the epidermis layer plateaued when the Young's modulus of the dermal papillae was lower than 0.04 MPa, as indicated by the material property sensitivity analyses. Indeed, these honeycomb-like micro-structures, consisting of stratified epithelium and dermal papillae, have also been found in the paw pads of other digitigrade mammals, such as cats and leopards (Hubbard et al., 2009; Ninomiya et al., 2011), suggesting that this characteristic structure may be desirable for quieter and faster locomotion patterns.

The moderated GRF, due to the cushioning function, could lead to low mechanical stress in footpad tissues. This is supported by the FE simulation results in this study, which showed that the peak von Mises stress in the epidermis layer was noticeably decreased by the honeycomb micro-structure, especially at higher impact velocities. This is in agreement with a previous experimental study that suggested that a honeycomb-like structure is advantageous because it acts as a shock absorber during impact (Yamashita and Gotoh, 2005; Burlayenko and Sadowski, 2010; Qian et al., 2010). However, a closer examination of the stress distributions in the whole epidermis layer revealed that stress was decreased only in the dermal papillae, whereas stress was higher in the stratified epithelium due to the honeycomb structure. It appears that the structured layer provides an offloading mechanism by transferring the load to the hard stratified epithelium whilst reducing the stress in the soft dermal papillae. This leads to significantly lowered (∼1000 times lower) von Mises stress at the top surface of the epidermis layer (around 10^−4^ MPa) than in the uniform layer. It is evident that the honeycomb micro-structure, consisting of a hard stratified epithelium and soft dermal papillae, provides an excellent offloading function, which protects the soft materials in the dermis and subcutaneous layers, hence maintaining the structural integrity of the footpad.

In addition to the lowered GRF and tissue stress, the structured epidermis layer also provides increased vertical displacement, which tends to rise with increasing impact velocity, due to the soft dermal papillae embedded in the cell units. This is generally undesirable because large vertical displacement may lead to a long stance duration and a slow-moving speed during locomotion. Furthermore, a large vertical displacement may worsen the lateral stability of the stance. However, in this study the maximum displacement was only 0.23 mm at an impact velocity of 0.4 m/s; therefore, the effect of displacement on locomotion was very limited. In this study, the stratified epithelium and the dermal papillae were considered to be linear elastic materials; however, biological materials are non-linear and visco-elastic in nature. The viscosity and the increased Young's modulus due to the material non-linearity would help to attenuate the vertical displacement when the impact velocity increases. Moreover, the vertical displacement would be further dampened by the dermis and subcutaneous layers of the hydrostatic system, especially by the subcutaneous layer, which is composed of many small compartments that are considered to be many small hydrostatic systems (Pond, 1998; Ker, 1999; Chi and Roth, 2010).

### Conclusions

Paw pad micro-structure was investigated in German Shepherd dogs and Chinese Rural dogs via SEM and histological examination. Dynamic FE analyses were conducted to assess the biomechanical function of the honeycomb-structured epidermis layer. It was found that the paw pad mainly has three layers, and that all three layers work as a whole to ensure the structural integrity of the pad system to meet the biomechanical requirements of locomotion. The tough stratum corneum layer, stratified epithelium layer and dermal papillae make up the epidermis layer, which sustains the harsh ground-pad interactions, weakens the ground impact and offloads high tissue stress. The dermis layer and the subcutaneous layer, including the many small closed compartments in the subcutaneous layer, further moderate ground impact and tissue displacement by absorbing the impact energy. The dermis layer connects the epidermis layer and the ‘hydrostatic system’. Results of this study provide more insight into the biomechanical functioning of the digitigrade paw pads and may facilitate development of bio-inspired ground-contacting components of robots and machines as well as impact the design of footwear and orthotic devices.

## MATERIALS AND METHODS

### Ethical statement

This study was approved by the Institutional Review Board Committee of Jilin University, Changchun, China (No. 20140418).

### Histological examination

A total of six dogs were included in the study: two German Shepherd dogs, that had died of heart disease, were donated by the police dog training base of the Public Security Bureau of Jilin Province in Changchun City; and four Chinese Rural dogs, which were euthanized via administration of an overdose of sodium pentobarbital for an anatomy teaching laboratory at Jilin University. The German Shepherd and Chinese Rural dogs had no recorded histories of musculoskeletal disorders or disease and their feet were intact and healthy. After death, the forefeet were amputated and tissue samples were taken from the metacarpal pads and prepared by the Animal Experiment Centre of the Bethune School of Medicine at Jilin University. The metacarpal pad specimens were fixed in l0% neutral buffered formalin for 48 h and then cut into 24 1.5-mm-thick slices in the sagittal and transverse planes, six approximately 8 mm×8 mm×8 mm cubes, and six approximately 8 mm×8 mm×3 mm cuboids. The 1.5-mm-thick slices were washed in distilled water, dehydrated with alcohol and embedded in paraffin for 3 h at 60°C. The paraffin tissue blocks were cut into 5-μm-thick slices and then stained with haematoxylin and eosin and Masson's trichrome stain for histological examination (Ninomiya et al., 2011). The cubes and cuboids were washed in distilled water and air-dried. All cube and cuboid samples were mounted on aluminium stubs and coated with gold palladium for observation (the cubes were examined in the sagittal plane and the cuboids were examined in the transverse plane) with a scanning electron microscope (EVO 18, Carl Zeiss Microscopy GmbH, Jena, Germany).

### Finite element modelling

**Fig. 7.**
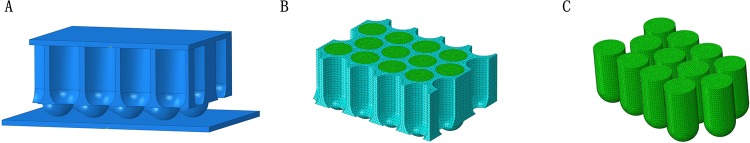
**The micro-scale finite element model of epidermis layer.** (A) Assembled model with the top plate and the ground surface. (B) Model of the stratified epithelium with embedded dermal papillae. (C) Model of the dermal papillae.

A micromechanical structured model of the epidermis layer of the paw pad was constructed to investigate the biomechanical function of this layer. A 1/400th portion of the layer was used to simplify modelling. The structured model consisted of a cube with twelve holes, arranged in a honeycomb and twelve cylinders (Fig. 7). The cube and cylinder were used to represent stratified epithelium structure and dermal papillae, respectively (Fig. 7B,C). The dimensions of the models are listed in Table S1. A rectangular plate was firmly connected to the top surface of the layer to represent the corresponding body mass (Alexander et al., 1986; Qian et al., 2014). Another fixed rectangular plate, placed beneath the tissue layer, was used to simulate the rigid ground surface. The three-dimensional (3D) geometry of the layer and plates was created using Solidworks software (Dassault Systèmes Corp., Waltham, MA, USA). The geometric parts were then imported and assembled using the FE modelling software ABAQUS (Dassault Systèmes Corp.). 3D tetrahedral meshes were used for the stratified epithelium structure and the papillae and 3D hexahedral meshes were used for the two plates. In total, 450,629 meshes were obtained for the whole model (Fig. 7).

In the FE simulation, the material properties of the stratified epithelium and dermal papillae were defined based on previous data (Cheung et al., 2005; Luboz et al., 2014) and were considered to be homogeneous, isotropic and linear elastic materials (Luboz et al., 2014). To simplify, the top and bottom plates were modelled as rigid bodies. The material properties and the element types of each part of the model are listed in Table 1. The dermal papillae were considered to be firmly embedded in the stratified epithelium structure without relative motion. The footpad-ground interface was defined as a contact surface with a frictional coefficient of 0.6 (Cheung et al., 2005). The top plate had a mass of 2.25 g, which was estimated from the mass of a representative cadaver dog and the percentage of the layer portion.

**Table 1.**
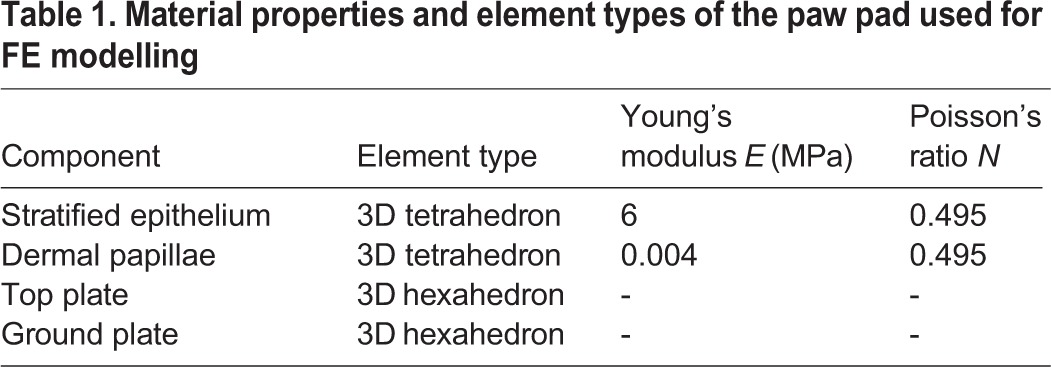
**Material properties and element types of the paw pad used for FE modelling**

### Dynamic simulation and sensitivity analysis

Dynamic FE simulations were conducted to analyse the biomechanical function of the structured epidermis layer in the stance phase of locomotion. The top plate, together with the stratified epithelium and the dermal papillae, was defined to contact the ground vertically with an initial velocity ranging from 0.05 m/s to 0.4 m/s, which was estimated from the metacarpal motion measurement data in the stance phase during normal walking in German Shepherd dogs. The dynamic responses of the vertical GRF, the vertical displacement of the top plate and the von Mises stress were all simulated and analysed at five different impact velocities: 0.05 m/s, 0.1 m/s, 0.2 m/s, 0.3 m/s, and 0.4 m/s.

To investigate the effect of the structured epidermis layer on biomechanical functioning, a uniform model was constructed, assuming that the entire layer was composed of the same homogeneous, isotropic and linear elastic material as the stratified epithelium. The same dynamic FE simulations were conducted in the uniform model using the same loading and boundary conditions as those applied to the structured model. In addition, material property sensitivity analyses were performed to examine the effect of Young's modulus of dermal papillae on the model prediction results. The material property analyses included four cases in which the Young's modulus (*E*, a measure of the stiffness of a solid material) of the linear elastic soft tissue of dermal papillae was changed to 0.0004 MPa, 0.04 MPa, 0.4 MPa and 4 MPa from the baseline value (0.04 MPa).

## Supplementary Material

Supplementary information
