# Supplementary material for: How does the canine paw pad attenuate ground impacts? A multi-layer cushion system
Source: Biol Open. 2017 Nov 23;6(12):1889–96. doi: 10.1242/bio.024828 (PMC5769641; doi:10.1242/bio.024828)
Supplement: Supplementary information [file biolopen-6-024828-s1.pdf]

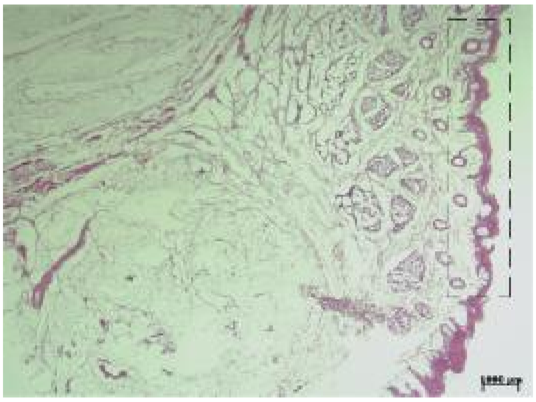

Fig S1: Transverse section near the side wall of the paw pad.
The boxed area is the side wall of the footpad that is not in direct contact with the ground surface.

Table S1 Detailed parameters of the models (mm)

The stratified epithelium structure model	
Length	3.15
Width	2.42
Total height	1.4
Height of the cube	1.1
Diameter of the hole	0.6
Height of straight hole	0.85
Distance between two holes	0.1
The dermal papillae model	
Diameter of the cylinder	0.6
Total height	1.15
Height of the straight column	0.85

Biology Open • Supplementary information
